# Evolution of the polycrisis: Anthropocene traps that challenge global sustainability

**DOI:** 10.1098/rstb.2022.0261

**Published:** 2024-01-01

**Authors:** Peter Søgaard Jørgensen, Raf E. V. Jansen, Daniel I. Avila Ortega, Lan Wang-Erlandsson, Jonathan F. Donges, Henrik Österblom, Per Olsson, Magnus Nyström, Steven J. Lade, Thomas Hahn, Carl Folke, Garry D. Peterson, Anne-Sophie Crépin

**Affiliations:** ^1^ Stockholm Resilience Centre, Stockholm University, SE-106 91 Stockholm, Sweden; ^2^ Global Economic Dynamics and the Biosphere Programme, Royal Swedish Academy of Sciences, SE-104 05 Stockholm, Sweden; ^3^ Anthropocene Laboratory, Royal Swedish Academy of Sciences, SE-104 05 Stockholm, Sweden; ^4^ Beijer Institute of Ecological Economics, Royal Swedish Academy of Sciences, SE-104 05 Stockholm, Sweden; ^5^ Potsdam Institute for Climate Impact Research, Member of the Leibnitz Association, 14473 Potsdam, Germany; ^6^ Fenner School of Environment & Society, Australian National University, Canberra 2601, Australia

**Keywords:** cultural evolution, social–ecological systems, participatory mapping, complex adaptive systems, evolutionary traps

## Abstract

The Anthropocene is characterized by accelerating change and global challenges of increasing complexity. Inspired by what some have called a polycrisis, we explore whether the human trajectory of increasing complexity and influence on the Earth system could become a form of trap for humanity. Based on an adaptation of the evolutionary traps concept to a global human context, we present results from a participatory mapping. We identify 14 traps and categorize them as either global, technology or structural traps. An assessment reveals that 12 traps (86%) could be in an advanced phase of trapping with high risk of hard-to-reverse lock-ins and growing risks of negative impacts on human well-being. Ten traps (71%) currently see growing trends in their indicators. Revealing the systemic nature of the polycrisis, we assess that Anthropocene traps often interact reinforcingly (45% of pairwise interactions), and rarely in a dampening fashion (3%). We end by discussing capacities that will be important for navigating these systemic challenges in pursuit of global sustainability. Doing so, we introduce evolvability as a unifying concept for such research between the sustainability and evolutionary sciences.

This article is part of the theme issue ‘Evolution and sustainability: gathering the strands for an Anthropocene synthesis’.

## 1. Introduction

The Anthropocene is a remarkable result of human cultural evolution [1]. Its deep cultural evolutionary roots can be seen through its connections to past human evolutionary transitions, including the evolution of symbolic language, cognition and social institutions and practices, such as agriculture [2–6]. These transitions have set in motion new trajectories through processes, such as multi-level selection and human niche construction, that can be self-reinforcing and have played important roles in the growing scale of human activities [7–10]. While this growth has delivered large increases in standard of living in many parts of the World, it also comes with its own new set of problems.

Today's globally connected systems are characterized by multiple interacting crises spanning the ecological, social, economic and technological domains [11–13]. The interconnected, global challenges of the Anthropocene lead to the question of whether we as humans could be on the verge of being, or already have become, locked into some form of undesirable trajectory with persistent crises and growing negative impacts on human well-being. Could the current Anthropocene trajectory be a trap that modern industrialized societies are naive to, not unlike seabirds feeding on deadly marine plastics, lacking the capacity to distinguish them from nutritious marine plankton? If so, how can societies leverage their collective cultural evolutionary potential to embark on a more sustainable trajectory [11,14–18]? Recent works indicate the important insights into similar questions from integration across sustainability and evolutionary science [1,19]. Although concepts such as traps and capacities for undergoing change are established in both fields, these concepts and questions have seen few attempts of integration [20–23].

In this paper, we use the concepts of traps and evolvability to seek further integration between evolution and social–ecological systems research. We first adapt the classic concept of evolutionary traps to a human and larger-scale Anthropocene context. We then present results from a participatory mapping and analysis of Anthropocene traps, including an assessment of their interactions, progression and severity. We end by exploring how the integration of the concept of evolvability with those of social–ecological resilience could help broaden and consolidate a research agenda on capacities needed to move out of trapped Anthropocene trajectories towards global sustainability.

## 2. Making sense of human evolutionary traps

**Figure 1.**
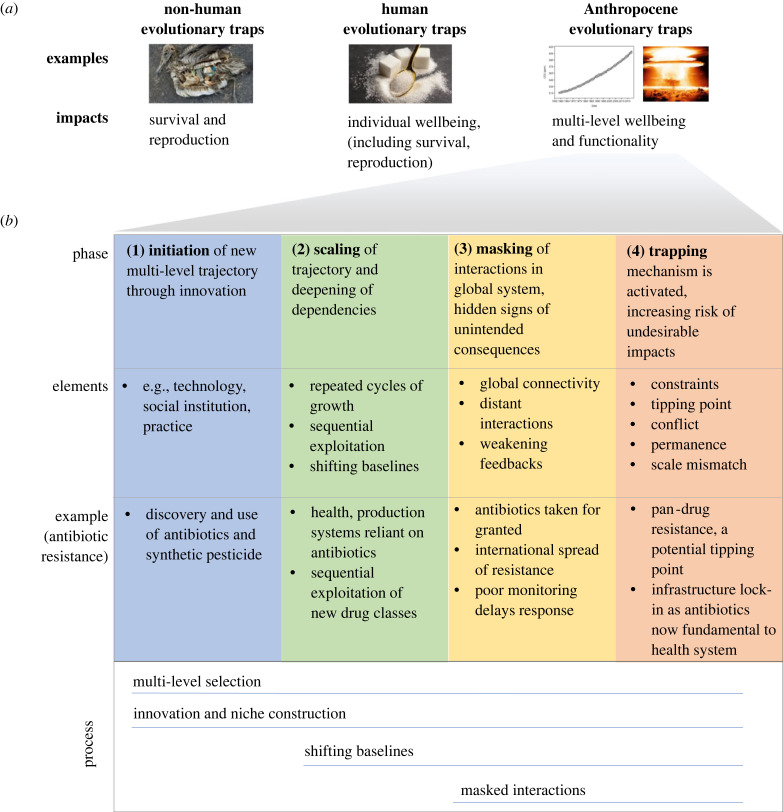
Anthropocene evolutionary traps. (*a*) Conceptualizations of evolutionary traps as applied to non-humans (traditional evolutionary traps, e.g. seabirds naive to marine plastics), individual humans (e.g. overconsumption of sugar in sugar-rich environments) and modern industrialized human societies in the Anthropocene (e.g. climate change and existential technologies such as nuclear weapons) (photo credits: albatross—Chris Jordan; sugar—fabrikasimf on Freepik; carbon dioxide concentration and nuclear explosion—public domain). (*b*) Phases in the evolution of Anthropocene traps and underlying principal processes illustrated with the example of the growing global challenge of antibiotic resistance [37].

The concept of evolutionary traps has been used almost exclusively for studying how non-human species respond to cues in anthropogenic environments [24–34]. Key examples include artificial human lights attracting insects, island species responding naively to the presence of introduced predators, and seabirds not being able to discriminate between the cues of marine plankton and marine plastics [34–36] (figure 1*a*). In the context of humans, evolutionary mismatch is a much more frequently used term compared with traps, especially in fields like evolutionary psychology and evolutionary medicine [38]. The differences in terminology between non-humans and humans could have two inadvertent consequences. First, the disuse of evolutionary traps in studies of human behaviour might inadvertently have prevented a deeper interrogation of the behavioural cues that maintain traps in human systems. Second, given how broadly used the concepts of traps are in systems-oriented sustainability science, such as social–ecological systems research, it might inadvertently have slowed down the interdisciplinary integration between evolutionary and sustainability sciences.

In classic evolutionary traps, organisms exhibit a preference for behaviour that lowers biological fitness through either survival or reproduction [33] (figure 1*a*, left). Applying the concept of evolutionary traps to humans immediately faces the challenge that humans are a highly cultural species with multi-level societies. It therefore requires an expanded concept that includes cultural and multi-level dynamics, with attention to key human capacities such as sense-making, reflexivity, forward-looking and anticipation [39].

We conceptualize human evolutionary traps at the individual level by looking at impacts on human well-being in addition to biological metrics of fitness (figure 1*a*, middle). Two major approaches to measuring individual well-being are objective and subjective measurement [40]. Objective approaches rely on indicators such as access to necessities like sanitation and healthcare, often collected in population-wide surveys. Subjective well-being relies on information expressed by the person in question about how content or able they are to fulfil life goals. Therefore, the human well-being approach considers impacts on a broad range of conditions of human individuals, from basic physical needs for survival to the subjective experience of life fulfilment.

We define Anthropocene evolutionary traps as phenomena manifesting at the global scale of human society, i.e. with dynamics occurring at least across multiple continents, causing one or more human practices to become maladaptive (figure 1*a*, right). Maladaptation becomes apparent through negative impacts on human well-being, from incremental to catastrophic. For Anthropocene traps, we are interested in large-scale regional or global trends in well-being metrics, as well as processes at higher levels of social organization and the environment that are critical for well-being. Relevant social processes include the functionality of institutions and organizations, production and provisioning of goods and services, as well as the infrastructure that facilitates coordinated movement of people, goods, energy and services. Relevant environmental processes include Earth system stability and the stability, provisioning and regulation of ecosystem services at large scales.

Our relatively broad approach to defining evolutionary traps in the human context should be seen in the context of continuing debates about how to conceptualize fitness for species with traits that are subject to intentional and cumulative cultural evolution [41–43]. We do not aim to solve those discussions here, but instead choose to be pragmatic in conceptualizing impacts of evolutionary traps in terms of human well-being. The advantage of this pragmatism is that we can investigate multiple evolutionary dynamics that interact to impact diverse aspects of human life. Another advantage is that it allows us to also take seriously the aforementioned human capacities, which means that humans can strive to make positive impacts on—sometimes normative—goals, such as well-being [16,43].

## 3. Methods: identifying and analysing Anthropocene traps

To identify and analyse a broad set of potential Anthropocene traps, we ran a set of participatory exercises, including seminars, workshops and questionnaires at the Stockholm Resilience Centre from 2020 to 2022. The initial exploration and identification of traps had three stages (electronic supplementary material, figure S1 for details). (1) Setting system boundaries. (2) Familiarization with evolutionary dynamics. (3) Identification of evolutionary traps. The insights from these activities provided the basis for the fourth stage of the study, assessment and analysis of traps (see §4. Analysis of traps).

1. *Anthropocene dynamics and system boundaries.* We started by surveying general dynamics of the Anthropocene system with the purpose of setting system boundaries for the subsequent exercise. A questionnaire asked employees at Stockholm Resilience Centre to open-endedly suggest important dynamics (processes) influencing or driving the Anthropocene trajectory as well as describe why these processes are important. We collected 61 processes and dynamics from 28 respondents. From this survey, it was clear that the scope of our further assessment explicitly needed to consider the technological, social and economic domains of the Earth system, including human behaviour, as well as biodiversity and ecosystems.
2. *Establishing a shared understanding of evolutionary dynamics.* As the experience of participants with evolutionary dynamics varied greatly, we sought to create a common understanding of core topics through four seminars with 12 internal and external speakers presenting conceptual, theoretical and empirical work relating to evolution in the Anthropocene. In addition to internal presentations by the authors of this paper regarding previous and ongoing work, external speakers included Timothy Lenton, David Sloan Wilson and Jeroen van den Bergh, covering systems perspectives, multi-level selection and evolutionary economics.
3. *Identifying Anthropocene traps.* With system boundaries set and an enhanced understanding of key concepts, we proceeded to solicit written suggestions for Anthropocene traps from a focus group of 10 participants (all authors) who had participated in the previous activities. These suggestions were then subject to common scrutiny and consolidation through two half-day workshops. The first workshop included eight participants from the focus group and aimed to review the initial set of traps, consolidating them into comparable groups of similar topical granularity as well as allowing time to think about traps that had not been identified in the written solicitation. The second workshop comprised 14 participants, including some not involved in suggesting initial traps, who served as scrutinizers. The workshop focused on review and agreement of proposed criteria for defining traps, establishing the final set of traps and a general model of how traps evolve.

We set three criteria for identifying a phenomenon as a potential Anthropocene trap: (1) that it can be described as evolving from an initially adaptive process; (2) that it, at the global level, shows signs of undesirable impacts on human well-being *or* has been hypothesized to show such signs in the future; (3) that it has a trapping mechanism that makes it harder to escape from negative impacts once this mechanism is activated.

## 4. Analysis of traps

Based on knowledge gained from the above activities, the identified 14 traps (table 1; details in electronic supplementary material, table S1) were subjected to further assessment by three authors (P.S.J., R.E.V.J. and D.I.A.O.), including data analysis, literature review and internally cross-validated expert opinion with input from the rest of the author group. As some traps describe general dynamics that can apply across a variety of topics and sectors, guided by author expertise and availability of evidence, we selected more concrete indicators that we could use for the assessment (table 1). Our assessment is summarized in table 1, reported in full in electronic supplementary material, table S1, and supported by documentation in electronic supplementary material, tables S2 and S4. Our analysis of the 14 traps proceeds as follows. We assess whether traps are growing as phenomena. We group traps based on their connection to evolutionary theory (electronic supplementary material, table S3) and describe trap interactions [44] (electronic supplementary material, table S4). Finally, we formulate a conceptual model for how traps evolve, and subsequently use this model to assess the current phase and severity of the 14 traps (electronic supplementary material, tables S1 and S2). The specific procedures involved in each of these analyses are described in the respective sections in this article.

**Table 1.** Anthropocene traps. Description and main dynamics are provided for the 14 traps together with indicator(s) used to identify the current phase (1, initiation; 2, scaling; 3, masking; 4, trapping) and trend (‘+’ growing, ‘±’ mixed). Phases are defined in §6 and figure 1*b*. The trapping column lists the mechanisms leading to entrenchment in the final phase (constraints, tipping, conflict, permanence, scale mismatch).

trap	description	indicator	trapping mechanism	phase	trend
*global traps*					
1. simplification	increasing specialization produces simplified sub-systems that are vulnerable to shocks	production ecosystems	constraints	4	+
2. growth-for-growth	institutional lock-ins drive pursuit of growth at the cost of well-being	well-being decoupling	constraints, conflict	4	+
3. overshoot	continued material growth leads to overshoot of Earth system tipping points	climate change	tipping point	4	+
4. division	unstable selection for global human cooperation increases risk of international conflict	international conflicts	conflict	3–4	±
5. contagion	global connectivity increases the risk of large-scale contagion, e.g. of infectious diseases	pandemic events	constraints	4	+
*technology traps*					
6. infrastructure lock-in	complex material infrastructure becomes maladaptive, e.g. owing to sunk costs	fossil fuel infrastructure	constraints	3–4	±
7. chemical pollution	capacity to produce complex or persistent compounds that can cause long-term harm to humans and ecosystems	assessment deficit	permanence, tipping point	4	±
8. existential technology	technological arms-races drive the evolution of existential technology, such as weapons of mass destruction	nuclear weapons	permanence	4	±
9. technological autonomy	reliance on automation can backfire if systems become misaligned to human needs	AI and robotics	permanence	2–3	+
10. dis- and misinformation	digitalization can amplify spread of mis- and disinformation e.g. destabilizing democracies	post-truth politics	permanence, conflict	3–4	+
*structural traps*					
11. short-termism	favour of short-term over long-term benefits reinforces other traps and promotes conflict	short-term growth focus	scale mismatch	4	+
12. overconsumption	separation of production and consumption facilitates overconsumption	footprints	scale mismatch	4	+
13. biosphere disconnect	separation of human settlements and ecosystems reduces awareness about their benefits	biosphere illiteracy	scale mismatch	3–4	+
14. local social capital loss	digitalization can lead to loss of local social capital through reduced interaction and echo chambers	social media polarization	scale mismatch	2–3	+

Rather than conclusive, this assessment should be seen as a first step in the development and application of a framework by a group of experts, as well as an invitation for further work and scrutiny. We acknowledge that the risk of traps may in some cases be unavoidable and has been a historical constant [9]. Yet, the global nature of the connected Anthropocene system warrants urgent understanding of these undesirable lock-ins. As any solution aimed at advancing sustainable development comes at the risk of initiating such trajectories, we end the manuscript by discussing capacities needed to navigate Anthropocene traps toward sustainability (§9).

## 5. Groups, trends and interactions of traps

**Figure 2.**
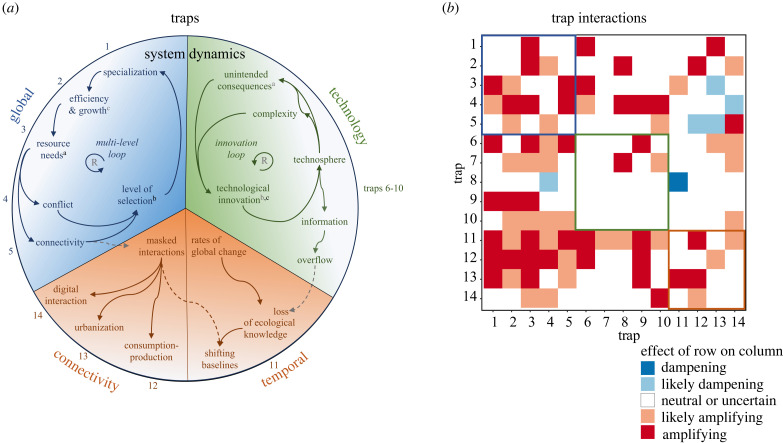
System dynamics and interactions of three major groups of Anthropocene traps: global (blue), technology (green), and stuctural traps (orange). (*a*) Systems diagram highlighting directed positive relationships between nodes as detailed in electronic supplementary material, table S3. Two reinforcing feedback loops are indicated with R and positive relationships across groups of traps are indicated with superscript letters (origin, black; receiver, grey) and dashed lined arrows. (*b*) A heatmap of the interactions between outcomes of the 14 proposed Anthropocene traps as described in electronic supplementary material, table S4, showing the effect of the trap listed in the row on that listed in the column.

We grouped Anthropocene traps in terms of underlying evolutionary dynamics based on their connection to three sets of theories, represented as causal loop diagrams in figure 2*a*. Construction of causal loop diagrams was based on the participatory activities and always validated by supporting references in the literature (electronic supplementary material, table S3). The first two sets are well established as evolutionary dynamics, namely multi-level selection for increasing levels of social organization and reinforcing dynamics of technological innovation [1,45–49]. A third set of theories relates to masked interactions and rates of global change and are well established in sustainability science, but less so as evolutionary processes, providing an opportunity for further integration of the two fields. Overall and based on selected indicators, we assess that 10 out of 14 traps are growing as phenomena, but these trends vary by groups of traps, as discussed below (table 1). We also find signs of widespread reinforcing interactions between traps, as described in §5d.

### (a) Multi-level selection and global traps

Multi-level selection of human cultural groups is widely theorized as a main driver of the trend toward higher levels of social organization, cooperation and global connectivity in the Anthropocene, as well as the increasing ecological footprint associated with this trend [1,47]. While this trend is often traced back to at least the agricultural transition, multiple subsequent transitions have reinforced this trajectory, e.g. when new sources of energy and new forms of mobility have been adopted [47,50–52]. At the global level, the lack of an out-group for cultural selection means that global cooperation is likely to be more unstable compared with lower levels [10,53].

We categorize five traps as global traps based on their connection to nodes in a five-step multi-level selection loop (figure 2*a* blue). The loop is initiated by selection for higher levels of social organization, which facilitates specialization and leads to increased efficiency and growth causing expanding resource needs. These needs can be solved through either conflict or cooperation (increasing connectivity of the system). Each downstream step in the loop has a trap associated with it. First, simplification and loss of response diversity constitute a risk of specialization (trap 1, simplification). Second, too much focus on efficiency and growth can lead to pursuit of growth at the cost of well-being (2, growth- for-growth). Third, continued resource extraction from expanding resource needs comes at the risk of ecological overshoot in the form of resource scarcity, environmental change and the crossing of ecological tipping points (3, overshoot). Fourth, the instability of cultural multi-level selection for cooperation at the global scale can lead to a trapped condition of global conflict (4, division). Fifth and finally, increased connectivity from global cooperation comes at the risk of contagion, such as in the spread of pandemic events or other shocks in the system (5, contagion).

**Figure 3.**
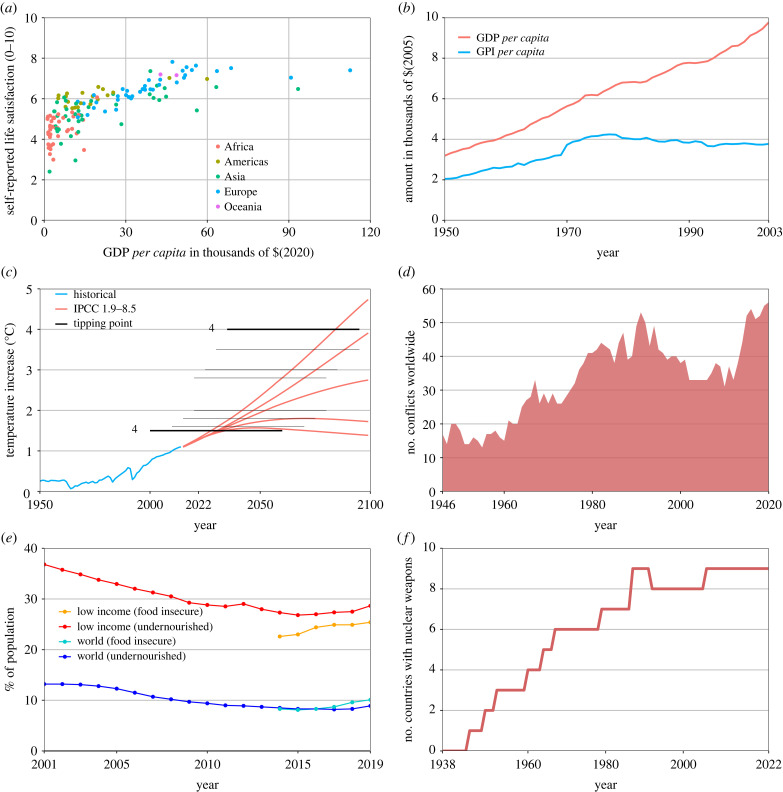
Trends in trapping mechanisms. Indicative trends of various Anthropocene trapping mechanisms and impacts. (*a*) Gross Domestic Product (GDP) *per capita* compared with self-reported life satisfaction, coded by continent [54,55]. (*b*) Changes in mean GDP *per capita* (at purchasing power parity) and mean Gross Progress Indicator (GPI) *per capita* over time [56]. (*c*) Temperature increase from pre-industrial levels over time from historical data (blue) and the five main Intergovernmental Panel on Climate Change (IPCC) scenarios (red), and median estimates (associated with high uncertainty ranges) of potential climate tipping point thresholds [57,58], where '4' indicates that four separate threshold medians are located around the thick black lines. (*d*) Number of conflicts (deaths ≥ 25 per year) worldwide over time involving at least one state-based actor [59,60]. (*e*) Percentage of the world population that is food insecure over time (Food and Agriculture Organization (FAO)) and percentage of the world population that is undernourished over time (World Bank) [61,62]. (*f*) The number of countries assumed to possess functional nuclear weapons worldwide over time [63,64].

Most global traps have shown growing trends towards the end of the recent 30-year period (figure 3; electronic supplementary material, table S2 for details and references). These trends can be summarized as: increasing shocks to simplified production ecosystems; increasingly speculative forms of global economic growth combined with global economic crises and growing inequality; worsened global environmental crises in the form of climate change and biodiversity loss; and increasing levels of tension between large nation states or regional political blocks and a rise of armed conflicts (figure 3*d*). Finally, there are some indications of a growth in frequency of global (re-)emerging infectious disease events, such as HIV, high-pathogenic bird and swine flu, antibiotic-resistant infections and COVID-19 [65]. But there is also uncertainty about the importance of monitoring bias in shaping these trends [66–68].

### (b) Innovation loops and technology traps

Niche construction describes the effects of organisms on the environment that influence selection pressures on these organisms [69]. Humans have a remarkable capacity for cultural niche construction, where cultural traits cause environmental change with selective effects on the same or other cultural traits [70]. Of specific relevance for the growing role of technology in the Anthropocene, humans can exhibit a preference for innovation of material technology as a problem-solving strategy for environmental change [1,46,70–72]. We identified five technology traps relating to an innovation loop consisting of two self-reinforcing dynamics (figure 2*a*, green). The first dynamic is that technological innovation results in unintended consequences, such as new environmental or social challenges that are often solved with new technological innovations [46]. The second dynamic operates through the transmission of technologies through cumulative cultural evolution, which means that there is a trend towards availability of more complex technologies to combine into and improve new technologies, a so-called ratchet effect [1,45,73].

In contrast to the global traps, the five technology traps do not map to individual nodes in the causal loop diagram, rather, they are outcomes of the overall dynamics. Among these, there are two phenomena that we consider more fundamental outcomes of the innovation loop, namely the risk of locking in to a material infrastructure, e.g. through sunk costs (trap 6, infrastructure lock-in) and the impacts on human health and the environment of new synthetic compounds and materials produced by the technology (7, chemical pollution). We consider the remaining three traps to be later-stage phenomena of advanced technology, namely the capacity of a species to exterminate itself with powerful technologies (8, existential technology), the risk of actions of increasingly autonomous technology not aligning with human goals (9, technological autonomy), and the risk of growing dis- and misinformation due to the exponential growth in information facilitated by digital information technology (10, dis- and misinformation).

Several technology traps show mixed trends in their selected indicators (electronic supplementary material, table S1). For infrastructure lock-in, there are large investments in and reduced costs of renewable energy, but also widespread dependence and continued investment in fossil fuel infrastructure [74–76]. Many forms of chemical pollution have decreased, but new forms are increasing [77]. For existential technology, there was a small increase in number of nuclear powers in the twenty-first century combined with a recent abandonment by some countries of disarmament treaties, yet a reduction of warheads. The two autonomous technology indicators are growing as investments in and new forms of artificial intelligence (AI) and robotics are on the rise, e.g. in terms of generative AI and self-driving cars [78,79]. The growth of digital information technology is also providing reach and speed to the spread of dis- and misinformation [80–82].

### (c) Scale mismatches and structural traps

Four out of the 14 traps do not map to the above well-established sets of evolutionary theory but to theories of shifting baselines and masked interactions in sustainability science [83–85] (figure 2*a*, orange). These dynamics are byproducts of the increased rates of global change and levels of connectivity that result from the multi-level and innovation loops. Specifically, rates of global change can result in loss of historical ecological knowledge (and social baselines), which together with information overflow from communications technology results in shifting baseline syndromes [86]. Similarly, masked interactions are a result of global connectivity and biased information transfer in global networks (e.g. supply chains) that reduce local signals for globally sustainable behaviours. We can conceptualize these dynamics as evolutionary processes by considering their impacts on socially transmitted information, a fundamental process of cultural evolution. Shifting baselines and masked interactions both influence what information is transmitted and reduce the adaptive value of that information through temporal and spatial scale mismatches, respectively.

Collectively, we refer to traps relating to scale mismatches as structural traps. Individually, we refer to traps from temporal scale mismatches as temporal traps and spatial scale mismatches as connectivity traps. Through its links to shifting baselines, a widespread focus on short-term economic growth and quick technological fixes was identified as the main temporal trap presenting a risk to long-term sustainability (trap 11, short-termism) [87,88]. Among connectivity traps, the separation of sites of consumption and production facilitated by global supply chains means that signals of environmental deterioration in production systems are weakened compared with a local system, increasing risks of overconsumption (12, overconsumption). Similarly, the reduced exposure to nature associated with urbanization comes at the risk of urban populations not being exposed to signals of environmental deterioration in remote systems that provide a benefit to them (13, biosphere disconnect). Finally, reduced face-to-face interactions in local communities resulting from digitalization and social media, which are currently increasing long-distance social interactions, could come at the risk of reducing local social capital and capacity for collective action (14, local social capital loss).

The four structural traps are all likely growing in importance (electronic supplementary material, table S1). In short, there is continued focus on short-term economic growth by national governments and businesses, despite some exceptions confirming the pattern [89,90]. Increasing material and ecological footprints indicate growing overconsumption facilitated by global supply chains [91]. More than half of the global population is now living in urban areas dominated by large spatial displacements of biosphere support functions and little green and blue space in many cities [92]. Finally, there are signs of increasing political polarization correlated with social media use in many countries [93].

### (d) Trap interactions

Traps interact at two levels: first, through the overall system dynamics that generate traps (figure 2*a*); second, through the outcomes of one trap on other traps (figure 2*b*). At the level of system dynamics, the multi-level selection and innovation loops often reinforce each other under joint dynamics sometimes referred to as sociocultural niche construction [1]. We identified three interactions between the loops where this reinforcement can occur (figure 2*a*, superscripts). First, resource needs in the multi-level loop are one type of unintended consequence in the innovation loop (^a^). Second, increasing levels of social organization and cooperation will often enable new innovative capacities [70] (^b^). Finally, technological innovation is one of the main ways through which specialization and increased efficiencies can occur and resource needs be addressed in the multi-level loop (^c^). As mentioned, the potential for structural traps is generated by the global changes in space and time set in motion by the multi-level and innovation loops. Yet there are even interactions between dynamics of structural traps where masked interactions help amplify the potential for shifting baselines and short-termism, as the local baseline is no longer a comprehensive indicator of ecological impact [94]. Thus, even in the presence of local monitoring for failures, such activities are no longer adequate.

At the level of trap outcomes, we assessed 182 pairwise interactions between traps (electronic supplementary material, table S4; figure 2*b*). Interactions were assessed using internally cross-validated opinion by the aforementioned three authors (P.S.J., R.E.V.J. and D.I.A.O.) based on insights gained through the participatory activities and using experts from the author group to help settle any disputes. Interactions were assessed on a five-point scale (figure 2*b*, colours). We estimate that 48% (87) have a net non-neutral effect (figure 2*b*; electronic supplementary material, table S4). Of these, 95% are amplifying (83/87) and only 5% dampening (6/87). This analysis reveals the systemic nature of Anthropocene evolutionary traps as well as the leverage in addressing root causes. It also means that addressing a couple of the traps could help alleviate several others. Foremost among such traps are global division, short-termism and overconsumption, as well as the growing concerns about technological autonomy, which all cause eight amplifying interactions.

## 6. A model of trap evolution

To assess the state of progression of traps, we first need a conceptual model of the general phases through which traps evolve (figure 1*b*; electronic supplementary material, table S2). Based on our dataset of 14 traps, we propose a model with four phases, namely (1) initiation of a new trajectory, (2) global scaling of the trajectory, (3) masked signs of negative impacts in the global system and (4) activation of trapping mechanisms and growing risks of negative impacts (figure 1*b*). Given the topic, it is unavoidable that this conceptual model of Anthropocene traps shares similarities with previous attempts of theorizing about general dynamics in social–ecological systems and the emergence of the Anthropocene explained by processes of cultural evolution. Examples of the former include the pathology of natural resource management and the adaptive cycle [95–97]. Examples of the latter include the work on sociocultural niche construction to explain the origin of the Anthropocene and evolution of unsustainability [1,19]. Our model of Anthropocene traps distinguishes itself from the former by explicitly focusing on the Anthropocene and the global scale and by integrating cultural evolutionary processes. Our model distinguishes itself from the latter work by explicitly focusing on the evolution of traps.

The model is a simple start for investigating Anthropocene traps and we anticipate new combinations of approaches will be needed for their more formalized modelling (box 1). Below we describe the processes involved in the four phases and we also illustrate the application of the four phases using the example of antibiotic resistance (figure 1*b*) [37]. We focus especially on the trapping phase as the processes of the first three phases have in part been covered as part of §5.

Box 1. **Approaches for modelling Anthropocene traps.**

From a systems perspective, Anthropocene traps are different from other traps in that they describe an evolving system trajectory rather than a rigid system state, as is usually the case for poverty traps, development traps and rigidity traps [98] (figure 1*b*). Understanding Anthropocene traps systemically may require applying more advanced methods and modelling approaches used in other fields of science for the analysis of complex adaptive and evolving systems or, perhaps even in some cases, entirely new modelling techniques. Traps are conventionally implemented in mathematical models as undesirable stable equilibrium states [99,100]. Anthropocene traps that lack adaptation may still be appropriately described as stable states. However, many Anthropocene traps, as they occur in complex adaptive and evolving systems, would be better described as stable (attracting) trajectories. For example, increasing resistance of pathogens against new antibiotics is not near any equilibrium of antibiotic resistance. Still, it is difficult to pull away from this accelerating, evolutionary ‘arms race’ trajectory owing to its inherent self-stabilizing feedbacks. In addition to characterizing whether a trajectory is a trap (its stability and resilience), other Anthropocene modelling challenges include searching for multiple kinds of attractors, dynamics in time and space, and fast and slow dynamic interactions, and mixing discrete and continuous-time processes.

### (a) Initiation and scaling

Processes of innovation and sequential selection and adaptation are major processes of the initiation and scaling phases, respectively. The initiation of trajectories through a social or technological innovation is described above as part of a multi-level loop. After this initiation, growing rates of environmental and social change increase the need for adaptation through further innovation, and multi-level selection increases connectivity and the establishment of global systems [3,101]. An important dynamic in these two phases that contribute to the early build-up of traps is that sequential selection will often address short-term, local and monitored (known) consequences, but less so undesired longer-term, spatially displaced and unmonitored global outcomes [95,101,102]. Two well-described and related forms of reinforcing dynamics in the scaling phase are sequential resource exploitation and innovation arms races. Illustrative cases of the former are sequential marine resources harvesting after collapsing stocks [103,104] and the sequential exploitation of new agricultural inputs, such as fertilizers, pesticides and machinery, to reduce labour costs [1,19,102]. An example of arms races is weapons arms races that occur in response to increasing threats (real or perceived), a process underlying the trap of existential technology, such as nuclear weapons [37,45,49,102].

### (b) Masking

In the masking phase, a diverse set of structural dynamics means that initial signs of global system failures, such as ecosystem service degradation or technological failures, are not addressed (figure 1*b*). First and foremost, among structural dynamics, global connectivity is causing interactions to become distant, e.g. through increasing trade, urbanization or information and communications technology [105,106]. A consequence of distant interactions is that people impact environments that they are not physically exposed to, and hence these impacts become increasingly masked [107,108]. Such local physical exposures have historically been strong selection pressures on human behaviour, and the removal of these increases the chance of maladaptive local behaviours at the scale of the connected system [109,110]. Cities, for example, offer little exposure to the ecosystems that provide most of the provisioning services of food, materials and textiles or the world's tropical forests and oceans, which harbour most of the biodiversity and make up the planet's largest carbon sink [111,112].

### (c) Trapping

In the fourth and final phase, trapping mechanisms are activated that make attempts to change trajectory exceedingly difficult and negative impacts on human well-being more likely. We identified five trapping mechanisms among the 14 traps: constraints; conflict; ecological tipping points with lagged and hysteresis effects; the permanence of cumulative culture; and scale mismatches. As shown in table 1, trapping mechanisms are unevenly distributed groups of traps. Technology traps are dominated by the permanence mechanism, which is a factor in four out of five traps. Structural traps are entirely characterized by scale mismatches as the main trapping mechanism. For global traps, trapping mechanisms are more diverse amongst global traps, where tipping points, constraints and conflict all feature as the main trapping mechanism. The five mechanisms are briefly introduced below.

First, constraints are a trapping mechanism well established in evolutionary science and with parallel concepts in social–ecological system research, such as rigidity traps [98,113]. Constraints in cultural evolution also arise because the lack of variation or the presence of maladaptive covariation between traits prevents an organism from adapting even in the presence of adaptive environmental cues [114]. Global challenges where such constraints are relevant include lack of response diversity in the global production ecosystem and association between old models of economic growth and international institutions [115].

Tipping points can be a powerful mechanism of luring a system into a new state or trajectory, especially when they involve temporal lags. When they involve hysteresis effects, crossing such ecosystem tipping points means returning to the previous state requires a disproportionate change to the system [116]. Committed emissions to future warming are prominently debated for climate change. In combination with tipping elements in the Earth system that, when crossed, further accelerate warming, such lags can potentially risk tipping the entire climate system to a new ‘hothouse’ state [57,117].

The conflict mechanism relates to the lack of a stable basin of attraction for global cooperation. As cultural multi-level selection has been a significant driver of increasing levels of human cooperation over time, the absence of this mechanism at the global scale could also form a trapping mechanism [10]. More generally, conflict trapping can occur when multiple actors are caught on local well-being peaks without the capacity to negotiate conflicting interests. Such dynamics can be seen among public as well as private actors in the form of ‘selfish states’ and ‘selfish corporations' that pursue short-term interests [90]. Ultimately, powerful actors with entrenched interests can keep global systems locked in to undesirable trajectories [118,119].

For technology traps, the permanence mechanism results from humans’ increasing capacity for cultural transmission, such that material technologies, once invented, are unlikely to go entirely extinct [45]. For example, the exponentially increasing storage capacity that digitalization has brought about means that uninventing technologies could become harder [120]. Finally, for structural traps, scale mismatches can be considered trapping mechanisms when temporal or spatial mismatches in cultural transmission become hard to realign*.* The irreversibility of scale mismatches could be seen as being of a softer (less material) character compared with, e.g. the permanence of cumulative material culture, but in practice such mechanisms could be just as consequential.

## 7. Current phases

The translation of phases into trap- and indicator-specific criteria is reported in electronic supplementary material, table S2. Applying these criteria and based on the available literature, we assess that 12 out of 14 traps could be in some stage of the trapping phase, of which four might only have progressed to the masking phase (table 1; electronic supplementary material, table S1). The remaining two of the 14 traps have not progressed further than the masking phase and might only be in the scaling phase. Using an ordinal scale, the average phase of traps is 3.67 and the median is 4 (min. 1, max. 4). Together with the overall growing trends in importance of the indicators, this assessment reveals a deepening and advanced status of many Anthropocene traps. Below we briefly highlight some more detailed findings for each of the three groups of traps.

Several of the global traps have likely activated, or are on the verge of activating, trapping mechanisms. Among these, institutional lock-ins to intensified production paradigms with low response diversity are a major constraint on making production ecosystems more resilient [121,122]. For ecological overshoot, the lagged temporal dynamics between emissions and warming mean that we are already committed to 1.5°C warming even though we are currently only at 0.8°C [123] (figure 3*c*). At the largest scale of cooperation for global sustainability, we see that the aims of individual nations are not aligned with the global level and many countries currently benefit from not cooperating for global sustainability [124]. Finally, COVID-19 has shown the large and robust constraints to managing globally connected systems and their vulnerabilities to pandemics [125].

For technology traps, we have seen the onset of trapping mechanisms relating to fossil fuel infrastructure lock-in, chemical pollution and nuclear weapons (electronic supplementary material, table S1). For example, sunk costs invested in fossil infrastructure are delaying climate action to a point of increasing negative climate impacts. Long-term effects and cocktail effects of pollutants are building up and poorly assessed [77]. And we have been trapped in a phase of existential risks from nuclear technology since the proliferation of nuclear arms. Thus, while not theoretically impossible, nuclear weapons technology has proven hard to uninvent [126]. In comparison, autonomous technology and dis- and misinformation are likely in an earlier phase of progression, as we currently see attention related to assessing and preventing some of their future risks. Thus, there is increasing attention to risks of AI and robotics; however, signs of many looming system failures may currently be masked. Despite attention to the regulation of digital information technology to prevent spread of dis- and misinformation, such efforts currently look unlikely to succeed in the short term.

Two of the structural traps appear to exhibit scale mismatches that are very hard to realign (electronic supplementary material, table S1). For short-termism, there are signs of a lock-in to harmful short-term growth strategies driven by a mismatch between individual benefit and collective harm [89,90,127,128]. For overconsumption, there are few signs of decoupling between economic growth and consumption-based footprints and few signs of reduced consumption in high-income countries, indicating large difficulties in aligning local consumption with its global consequences [129]. In urban areas, there are some signs of a counter movement to the risks of a growing biosphere disconnect, e.g. through coalitions of cities, such as C40 seeking to provide leadership on climate change. Yet it is still uncertain if such initiatives can make cities act as overall regenerative global agents of the Earth system and distant ecosystems. For social media polarization, we may not yet be trapped, but regulation is proving hard. The question is whether globally connected societies can be aligned with the building of local social capital [130,131].

## 8. Severity of traps

As the trapping phase progresses, negative impacts on human well-being are more likely to become obvious and widespread. Evolutionary biology often distinguishes between two levels of severity of traps, severe and equal-preference traps. Severe traps have absolute negative impacts on the population and more readily lead to a population decline, whereas an equal-preference trap is harder to detect in terms of population-level impacts without an experimental set-up [34]. Given the obvious limitations of this approach for Anthropocene traps, we suggest focusing on domains of well-being affected. For example, negative impacts from the simplification of food production ecosystems can transfer from the realm of food insecurity (the subjective assessment of being food insecure) to undernourishment, the state of not getting enough calories, which in turn can have consequences, such as stunting, wasting and ultimately increasing child mortality. Applying this approach, we used generally available global datasets and reports to assess current trap severity.

Several observed negative trends in human well-being are likely produced by the interaction between global traps. This includes the recent increases in food insecurity and undernourishment in low- and middle-income countries (figure 3*e*) [132]. These are major impacts of the simplification trap that are worsened by, e.g., ecological overshoot and growth-for-growth [132,133]. For the growth-for-growth trap, the cross-country pattern of stagnation in subjective well-being with economic growth could indicate a relatively mild trap (figure 3*a*), but the severe human costs of shocks from economic crises [134–139], the above trends in food insecurity, and the indirect effects through ecological overshoot indicate more severe impacts. For ecological overshoot, some of the most tangible indicators of current severity include health and economic impacts of extreme weather events and long-term droughts [140–143], as well as impacts from ecosystem collapses and diminished ecosystem services [144,145]. For global division, current severity is indicated by hardship due to international economic conflict as well as through lost livelihoods (e.g. from forced migration), disability and deaths from armed conflicts [59,60,146]. There are also indirect costs of lack of collective action in addressing global and social environmental challenges [147–149]. Finally, health impacts of recent pandemics, HIV, antimicrobial resistance and COVID-19, as well as indirect impacts from efforts to manage these pandemics are relevant indicators of the severity of the contagion trap [65,150–153].

The severity of technology traps is currently most clearly illustrated for fossil fuel infrastructure, which is one of the major contributors to the negative impacts of air pollution on human health as well as indirectly through climate change [141,142,154–156]. While many chemical pollution effects are hard to discern, recent work has quantified impacts relevant for antibiotics in terms of mortality from antibiotic resistance to more than one million extra deaths [157]. Existential technology exhibits peculiar severity dynamics as it may be one of the most irreversible traps, yet so far, its catastrophic impacts have mainly manifested locally, with risks of larger-scale impacts looming as demonstrated by Russia's threats during the invasion of Ukraine [158–161]. Thus, another dimension of well-being that is affected by existential technology is in terms of instilling fear and contributing to deadlocks in solving conflicts between nuclear powers. Many impacts of AI are largely unknown, but have been investigated in terms of amplifying social biases [162,163]. Finally, impacts of post-truth politics can be measured in terms of individually harmful behaviours (e.g. anti-vaccine deaths during COVID-19), social unrest and delayed collective action.

The severity of structural traps can be assessed at two scales, first, in terms of impacts on well-being of the communities involved in the trap, and second, as the contribution of the structural traps to larger ongoing global and technology traps. For the latter, several structural traps (short-termism, overconsumption and biosphere disconnect) interact with the ecological overshoot, growth-for-growth and likely several technology traps (figure 2*b*). The loss of local social capital and political polarization contributes both to the dis- and misinformation trap and potentially also to global division, as national tensions can affect the ability of actors to engage in international compromises [86,164].

Many direct impacts of structural traps are poorly quantified, but relevant variables can be identified for further study. For short-termism, shifting baselines involves loss of historical ecological and cultural knowledge, which can impact subjective aspects of well-being such as identity and belonging (sense of place), but also loss of adaptive capacity [85,165–167]. The severity of the overconsumption trap can be measured e.g. through polluting activities, loss of ecosystem services and poor labour conditions in remote production sites. Relevant impacts of the biosphere disconnect trap are urban impacts of climate change and disruptions to biodiversity ecosystem services, including through pandemics. For social media polarization, there are several studies highlighting the correlation between mental health issues and social media use [168–170].

## 9. Navigating traps: evolving toward sustainability

In studies of non-human evolutionary traps, organisms can escape through adaptive genetic responses or some form of learning [171]. In humans, the relevant question is how humans can intentionally evolve culturally through socially and ecologically inclusive processes to pursue this relationship [11,16,43,172].

Evolvability is a term historically used in parts of evolutionary biology to reflect the ability of an organism to evolve in response to new circumstances [20,173,174]. We propose that in the context of sustainability for culturally complex species such as present-day humans, evolvability can be thought of as a set of cognitive, social and social–ecological capacities [22,114,175]. Defined as such, there is much activity in both cultural evolution and social–ecological systems research aiming to understand human evolvability. In cultural evolution, there is work on intentional cultural evolution [11,16,43,172] and increasing emphasis on understanding some of the processes that underlie long-term evolvability, such as the generation of novelty through innovation [20,176,177]. In sustainability science, an influential school of thought on evolvability is that of social–ecological resilience as capacities of persistence (absorbing shocks), adaptive capacity (responding to change) and transformability (changing the identity of a system) [22,23,178,179].

For the question of whether modern societies can leverage their cultural evolutionary potential to avoid severe Anthropocene traps and move towards global sustainability, we define global sustainability as a trajectory or state where humans improve well-being through conscient protection and stewardship of a socially inclusive and biodiverse planetary system [11]. In evolutionary terms, such a conscient transition toward integration with a planetary system would be unprecedented and has been proposed as a new aeon, the Sapiezoic [180]. It would distinguish itself from previous events where species had revolutionary impacts on planet Earth, such as the great oxygenation event that caused a mass extinction of anoxic life forms, not only by being conscient, but also by preserving an existing biota and its functions [181,182].

Based on our above analysis of Anthropocene traps, we here suggest five overarching aspects of evolvability that will be important for navigating toward global sustainability. These are (a) the capacity to recognize traps and set goals for evolving out of them, (b) the capacity to learn about where we are in relation to these goals and what steps are needed to approach them, (c) the capacity to reorganize and innovate, (d) the capacity to be prepared for and respond to surprise, and (e) the capacity to navigate conflict. The first and second capacities relate to recognizing current and potential future Anthropocene traps and understanding how to get out of them; the third and fourth capacities relate to implementation of such strategies; and the fifth capacity relates to the ability to do this at a global level. Here we briefly discuss and give examples for each of them.

### (a) Recognizing traps

At the global level, there are clear signs of increased awareness of some pressing or looming Anthropocene traps expressed by their inclusion in global policy frameworks. Increasingly integrated agendas like the Sustainable Development Goals (SDGs) are an encouraging sign of efforts towards setting goals for global sustainability [183]. There is, however, room to improve. For example, the SDGs have been criticized for being short-term goals and for not delivering a set of clear priorities to the 17 goals [184]. This comes at the risk of countries cherry-picking goals and e.g. mixing concepts of relative sustainability (improvement) and absolute sustainability (respecting certain thresholds). In addition to these risks, inaction is not sanctioned, which could explain slow progress on many goals [124].

### (b) Measurement and foresight

Basic but sufficiently accurate measurements that capture ongoing social and environmental change are essential to detect and act on traps. Measuring human well-being and natural and social capital is essential here. Recent years have seen increased activity in this field, such as the design of new inclusive metrics of economic growth and growing global databases of human well-being indicators [185,186]. However, the Anthropocene system is still operated by conventional metrics and institutions [187].

Increasing human capacity for foresight is vital to avoid undesirable technology traps. Foresight involves enhancing our aptitude to predict both Earth system and social dynamics and depends on the ability to learn from available information gathered through measuring and monitoring what matters. Current measurements and metrics may fulfil some of the current needs. However, increasing human capacity to foresee traps in the future will require metrics that truly encompass human well-being's dependence on the biosphere [11].

### (c) Reorganizing and innovating

Phasing out and reorganizing institutions and sectors that are locking us in to or toward trapped conditions constitute an essential capacity to start moving along desired pathways. For example, the Bretton–Woods institutions have focused excessively on economic growth measured as GDP rather than growth in well-being. Thus, they may not function as intended from the beginning [188]. To move towards a more sustainable economic growth model, these institutions' traits will have to be reconfigured. In addition, generating new solutions can complement the reconfiguration of existing ones. A key innovative capacity for sustainability will be to deploy new social and nature-based practices and large-scale solutions [189,190] that help reduce Anthropocene risks [191,192]. Enhancing this capacity should be compared with the lock-in risks involved in geoengineering based mainly on material technologies that can potentially amplify technology traps [193].

### (d) Being prepared for the unknown

While enhanced abilities to reconnect, predict and innovate will be necessary, human societies must always be prepared for surprises in the form of unknown unknowns [194]. Maintaining the capacity to adapt to future surprises requires bolstering diversity in the form of variation and redundancy [115]. Modular and re-usuable designs ensure that institutions and infrastructures can be rearranged quickly in response to unexpected change. It is also essential to nurture methods to identify timely and appropriate responses despite these uncertainties and surprises. Such methods build on embracing uncertainties and being prepared to find sufficient evidence, prioritize no-regrets policies and get the big picture roughly right by abstracting from irrelevant details [195].

### (e) Navigating conflict

As global division can potentially reinforce many other traps, a key capacity for human evolvability is to navigate such conflicts between levels and domains of social organization, from the global to the individual. Here human ability to work with each other with diplomacy and bridging of perspectives will be necessary. While these abilities work well locally, they can be more challenging to maintain at larger scales and require the ability to reconcile sometimes quite diverse perspectives and needs.

The climate negotiations are a prime illustration of the significant conflicting incentives between mainly high-, low- and middle-income countries. The perceived short-term benefits of quickly addressing climate change are minor for low- and middle-income countries if they prevent economic and well-being growth, and not even high-income countries are on target to achieve these goals [196]. In parallel with trying to solve these conflicts at the highest level, coalitions of like-minded private and public actors can work to increase incentives for global sustainability action [197–198].

To facilitate these overarching capacities, we propose that a key capacity in evolving for sustainability will be to collectively imagine new futures. Creating common narratives and stories and local versions of these here will be necessary [43]. In the past, cultural multi-level selection theory has identified the perception of a common enemy as important for collective action [109]. In the Anthropocene, narratives will have to recentre on both a common friend and enemy—the common friend being Earth and its capacity to support life. The common enemy could well be the hostility of outer space and the difficulty of surviving in numbers anywhere else than within the biosphere [11,18,200].

## Data Availability

All the data supporting the findings of the paper are presented in the supplementary material and the tables therein [201].
